# Transcriptomic Analysis, Motility and Biofilm Formation Characteristics of *Salmonella typhimurium* Exposed to Benzyl Isothiocyanate Treatment

**DOI:** 10.3390/ijms21031025

**Published:** 2020-02-04

**Authors:** Tong-Xin Niu, Xiao-Ning Wang, Hong-Yan Wu, Jing-Ran Bi, Hong-Shun Hao, Hong-Man Hou, Gong-Liang Zhang

**Affiliations:** 1 School of Food Science and Technology, Dalian Polytechnic University, Dalian 116034, China; ntx156560120@gmail.com (T.-X.N.); wxn1531972535@gmail.com (X.-N.W.); bijingran1225@foxmail.com (J.-R.B.); houhongman2011@hotmail.com (H.-M.H.); 2 Graduate School of Environmental and Life Science, Okayama University, Okayama 700-8530, Japan; wuhongyan1908@hotmail.com; 3 Department of Inorganic Nonmetallic Materials Engineering, Dalian Polytechnic University, Dalian 116034, China; haohs@dlpu.edu.cn

**Keywords:** *Salmonella typhimurium*, benzyl isothiocyanate, transcriptome, motility, biofilm

## Abstract

*Salmonella typhimurium* (*S. typhimurium*) is a common foodborne pathogen that not only causes diseases and contaminates food, but also causes considerable economic losses. Therefore, it is necessary to find effective and feasible methods to control *S. typhimurium*. In this study, changes in *S. typhimurium* after treatment with benzyl isothiocyanate (BITC) were detected by transcriptomics to explore the antibacterial effect of BITC at subinhibitory concentration. The results showed that, in contrast to the control group (SC), the BITC-treated group (SQ_BITC) had 197 differentially expressed genes (DEGs), of which 115 were downregulated and 82 were upregulated. We screened out eight significantly downregulated virulence-related genes and verified gene expression by quantitative Real-time Polymerase Chain Reaction (qRT-PCR). We also selected motility and biofilm formation to observe the effects of BITC on the other virulence related factors of *S. typhimurium*. The results showed that both swimming and swarming were significantly inhibited. BITC also had a significant inhibitory effect on biofilm formation, and showed an effect on bacterial morphology. These results will be helpful for understanding the mechanism of the antibacterial action of BITC against *S. typhimurium* and other foodborne pathogens.

## 1. Introduction

In recent years, with increasing numbers of new foodborne diseases caused by microbial contamination, consumers and the food industry have begun to focus on food safety [1]. *Salmonella typhimurium* (*S. typhimurium*) is a common, gram-negative, food-borne bacterium with flagella [2]. It is widely found in poultry, animal husbandry, and many kinds of foods and animal feed all over the world and has an extremely important economic impact on food safety and health [3]. According to the previous research, when *S. typhimurium* causes fatal infections in mice, multiple virulence genes are required, and the pathogenicity of the bacteria is closely related to its locomotion and membrane formation abilities. The components of the outer membrane (proteins and lipopolysaccharides) as well as the pili and flagella, play important roles in the colonization and systemic infection in animal and human hosts of *S. typhimurium* [4,5]. Once attached to an abiotic surface, planktonic bacterial cells gather in hydrated extracellular aggregates to synthesis the proteins, polysaccharides, and nucleic acids to form biofilms [6]. Biofilms protect bacteria in a growth pattern that allows them to survive harsh environments [7], and bacteria that produce biofilms are more resistant to antibiotics. Study of the motility and biofilm formation of *S. typhimurium* helps researchers to efficiently design and optimize the beneficial properties of various mechanisms of new anti-infective drugs [8]. *S. typhimurium* infections are so deadly that more than a million people are treated each year in the United States alone. In consequence, the economic impact of *S. typhimurium* infection on food safety and health has been a driving force in the development of new antimicrobial agents [9].

In the past, people have tried to solve this problem using synthetic chemical preservatives. However, the adverse effects on humans from the overuse of chemical preservatives have far exceeded those of the pathogenic microorganisms themselves [10]. Therefore, we urgently need to replace synthetic preservatives with natural antimicrobials. Benzyl isothiocyanate (BITC) which is extensively found in senvy, pilu oil, cruciferous plants and papaya seeds [11], has been shown to exert potential health benefits to humans. According to the recent studies, BITC shows excellent therapeutic effects on cancer by reversing or blocking the proliferation of DNA-damaged precancerous cells [12,13,14]. In addition, BITC antibacterial mechanisms in foodborne bacteria have been widely studied. For example, the biofilm integrity, bacterial morphology, membrane potential, ATP, and membrane hydrophilicity of bacteria treated with BITC have been investigated [15,16,17]. However, the mechanism of antibacterial activity of BITC on *S. typhimurium* is still unclear, and further studies are needs. 

In the last decade of rapid technological development, transcriptome technology has become an essential tool in the biological sciences [18,19]. Transcriptomic analysis is widely used because it can comprehensively evaluate differential genes and enrichment pathways in samples. The transcriptomics has been applied to comprehensively analyze *S. typhimurium* for exploring bacteriostatic mechanisms [20,21]. For example, the effects of cranberry extract and antimicrobial proteins on the growth rates and transcriptomics of *S. typhimurium* were investigated. The expressions of related genes, such as those involved in iron acquisition, flagella, energy metabolism, pathogenicity, virulence islands and the cell membrane, were significantly downregulated after bacteriostatic treatment in *S. typhimurium* [22,23]. According to Song et al. and Wang et al. [24,25], the transcriptome was used to explore the mechanism of antibacterial activity of BITC on *Vibrio parahaemolyticus* and *Staphylococcus aureus*. It was shown and proved that BITC had a good bacteriostasis effect on these two food-borne pathogens. However, studies on the mechanism of BITC action on *S. typhimurium* by transcriptome detection have not been reported.

Therefore, to study the mechanism of BITC action on *S. typhimurium*, we conducted an in-depth analysis at the transcriptome level. In order to investigate the bacteriostatic mechanism of BITC, the differential expressed genes related to virulence were screened out through the transcriptome results. The expression of genes was further verified by quantitative Real-time Polymerase Chain Reaction (qRT-PCR). Additionally, the effects of BITC on the motility and biofilm formation of *S. typhimurium* were studied, and the mechanism of BITC action on *S. typhimurium* was discussed from the molecular and phenotypic levels.

## 2. Results

### 2.1. Antibacterial Tests 

According to the minimal inhibitory concentration (MIC) determined by microbroth dilution, the MIC of BITC for *S. typhimurium* is 250 μmol/L. According to the results of the antibacterial test, the subinhibitory concentration of BITC in the subsequent experiments was determined.

### 2.2. Global Changes at the Transcriptome Level 

To analyze the effects of BITC on *S. typhimurium*, 1/4 MIC (62.5 μmol/L) was selected as subinhibitory concentration for the following studies. The transcriptome sequencing was performed on the RNA of the control group (SC) and the 1/4 MIC BITC-treated group (SQ_BITC). The product was purified by PCR amplification. Finally, six cDNA libraries were built. Each of the libraries was sequenced using Illumina HiSeqTM 2500/MiSeqTM. We acquired a total of 16.44 Gb of data, including 112,664,390 raw reads and 109,599,530 clean reads. The six cDNA libraries for SC and SQ_BITC samples generated 24,399,752, 21,013,012, 14,444,786, 17,951,308, 15,258,326, and 19,597,206 clean reads. An overview of the transcriptome assembly statistics is shown in Table 1. The error rate of single base location sequencing in all six groups was less than 1%. The Q20 and Q30 percentages were equal to or higher than 98% and 94%, respectively. The downstream analyses were based on good quality data of Q20 and Q30.

### 2.3. Analysis of Differentially Expressed Genes (DEGs)

**Figure 1:**
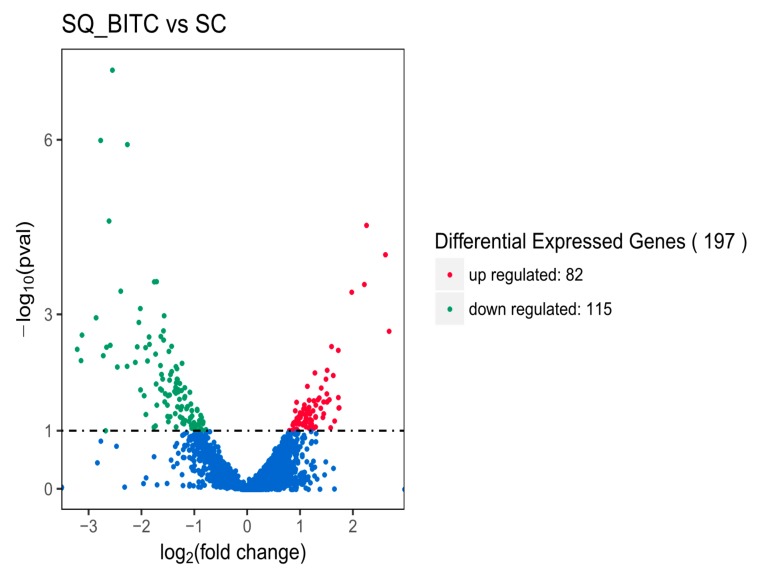
Volcano map of differentially expressed genes (DEGs) for SQ_BITC vs SC. Different colors (red, green, and blue) represent upregulated, downregulated, and no significant changes, respectively, in DEGs between the SQ_BITC and SC groups.

RNA sequencing technology was used to analyze SC and SQ_BITC samples to study the inhibitory mechanism of BITC on *S. typhimurium*. The analysis of DEGs was divided into three parts: volcanic maps, gene ontology (GO) analysis and Kyoto encyclopedia of genes and genomes (KEGG) signaling pathway enrichment analysis. Volcanic maps were used to show the overall distribution of the differentially expressed genes in the SQ_BITC and SC groups. The selection criteria were *P* < 0.05. A total of 197 important DEGs were identified in the experimental group. There were 115 downregulated genes and 82 upregulated genes (Figure 1).

**Figure 2:**
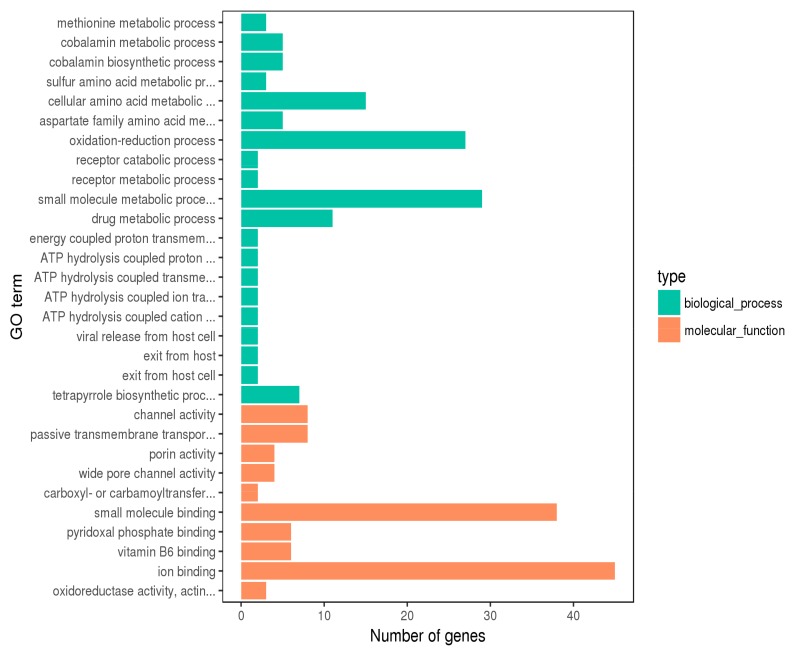
Gene ontology (GO) analysis of differentially expressed genes (DEGs) for SQ_BITC vs SC. The 30 GO terms with the most significant enrichment were shown.

Gene ontology is a classification system that includes molecular functions, biological processes and cell components. As shown in Figure 2, the distribution of differential genes in GO was used to explore the differences in physiological and metabolic functions and biological processes between the experimental group (SQ_BITC) and the control group (SC). According to transcriptomic data, 197 genes were annotated into different GO terms. The 30 GO terms with the most significant enrichment were selected and included 20 biological processes and 10 molecular functions.

**Figure 3:**
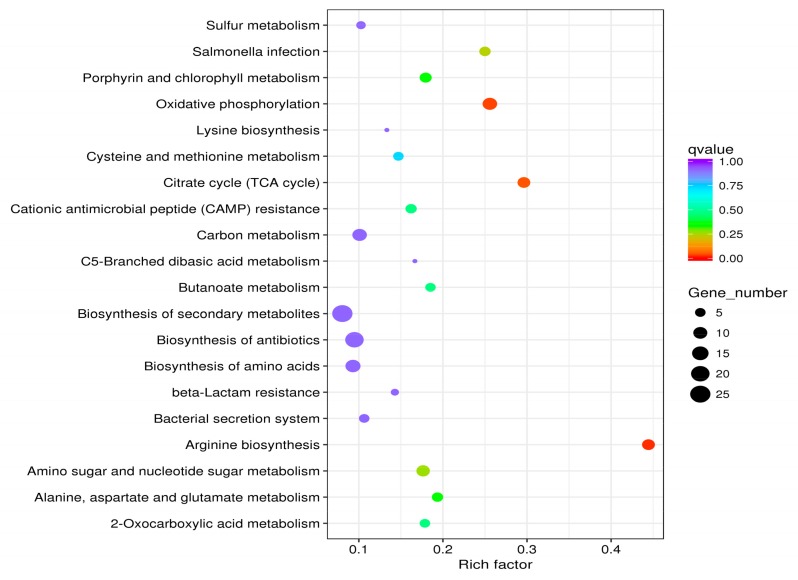
The most abundant pathways for a total of 197 differentially expressed genes. The size of the dot is proportional to the number of genes; the closer the *q* value is to 0, the greater the extent of enrichment.

Each biological function is completed through the cooperation of different genes. The most important biochemical or signaling pathways in which differentially expressed genes participate are determined by the significant enrichment of the pathway. Following GO analysis of the differential genes, KEGG signaling pathway enrichment analysis was performed. Compared with the control group, 197 differentially expressed genes were enriched for 61 pathways after the addition of 1/4 MIC BITC in the experimental group; the 20 most abundant pathways were selected, as shown in Figure 3. Arginine biosynthesis, oxidative phosphorylation, citrate cycle (TCA cycle), Salmonella infection and amino sugar and nucleotide sugar metabolism pathways were the most significantly enriched.

### 2.4. Expression of Virulence-Related Genes by qRT-PCR

**Figure 4:**
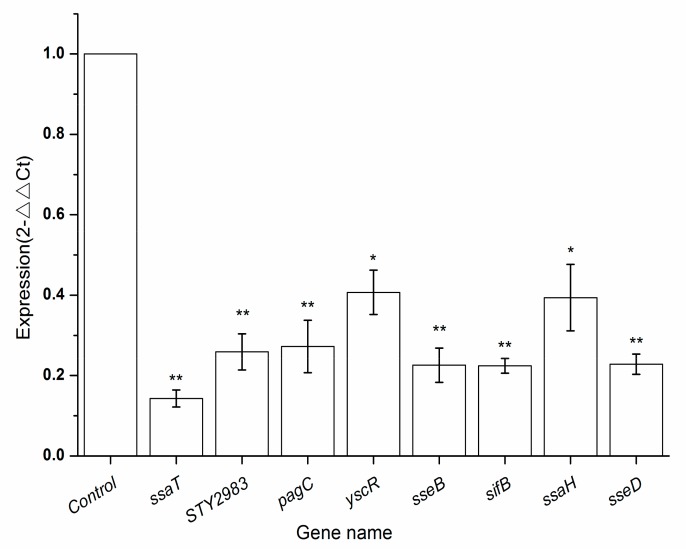
qRT-PCR validation of differentially expressed genes (DEGs). The relative expressions of *ssaT*, *STY2983*, *pagC*, *yscR*, *sseB*, *sifB*, *ssaH* and *sseD* were compared with *16S rRNA* in the control group. The bars are expressed as the means ± SD from three independent replicates. ‘*’ indicates significant differences (*p* < 0.05). ‘**’ indicates extremely significant differences (*p* < 0.01).

For gene expression, qRT-PCR is a powerful tool and the most commonly used method to confirm transcriptomic data and analyze genes. By screening out genes related to virulence through the transcriptome results, qRT-PCR can directly reflect the influence of BITC on the expression of its pathogenicity correlation genes, further verify the transcriptomic results. From the RNA sequencing data, virulence-related genes with significantly reduced expression were selected according to the transcriptomic results, including *ssaT*, *STY2983*, *pagC*, *yscR*, *sseB*, *sifB*, *ssaH*, and *sseD*, as shown in Table 2. The validation results of DEGs were consistent with the results of the RNA-seq analysis, indicating that RNA-seq successfully identified DEGs. Additionally, it was verified that, the expression of virulence-related genes decreased significantly in *S. typhimurium* by BITC treatment. The results are shown in Figure 4.

### 2.5. Motility of S. typhimurium Induced by BITC

**Figure 5:**
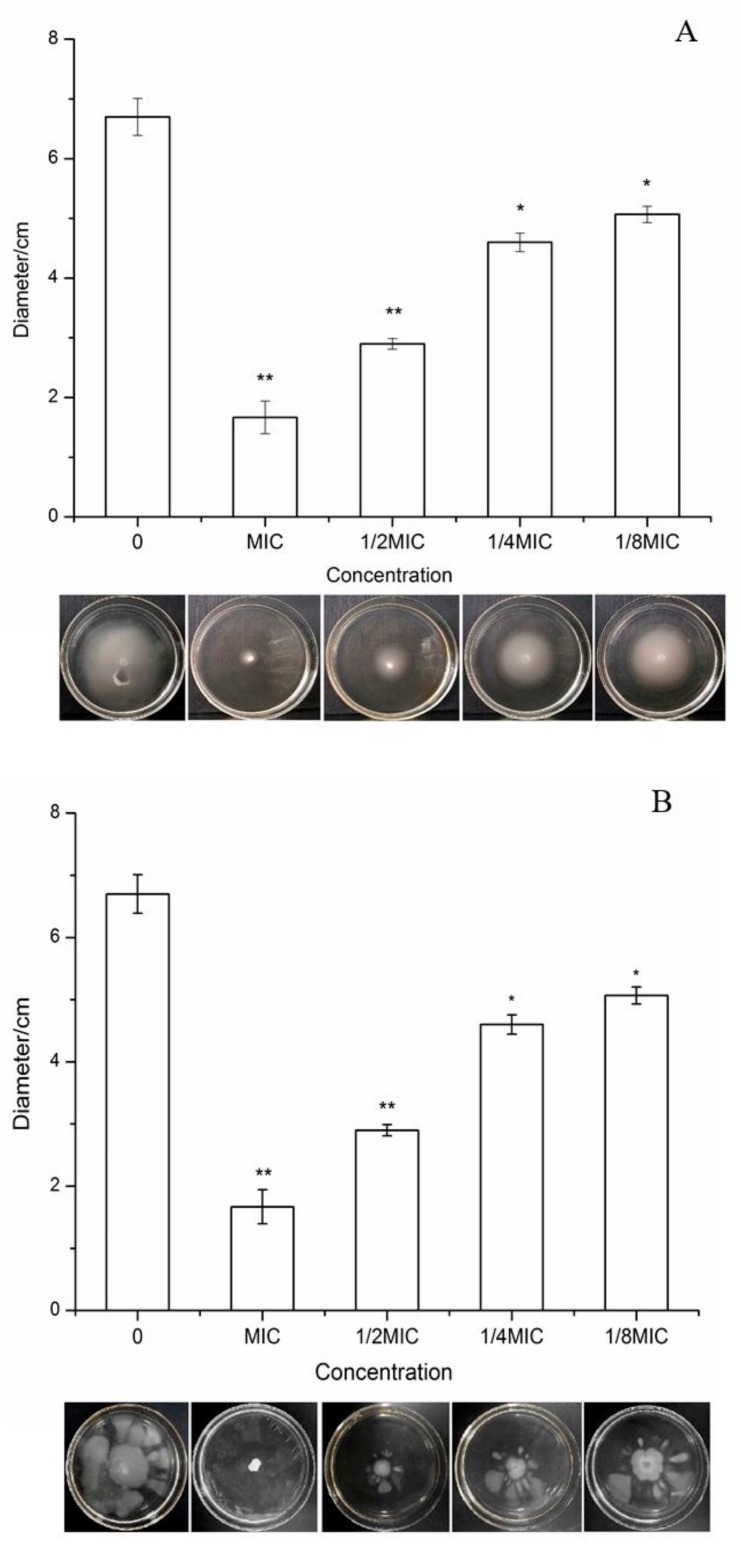
Inhibitory effects of BITC at different concentrations on the motility of *S. typhimurium*. The bacteria were treated with BITC at different concentrations (MIC, 1/2MIC, 1/4MIC and 1/8MIC) at 37 °C for 12 h (**A**) and 48 h (**B**), respectively, and the diameters were observed and measured. Figure A indicated the results of swimming and Figure B indicated the results of swarming. ‘*’ indicates significant differences (*p* < 0.05). ‘**’ indicates extremely significant differences (*p* < 0.01).

The motility of bacteria is related to the presence of flagella, which is closely related to the pathogenicity of bacteria. By observing the motility of *S. typhimurium*, we expected to explore the effect of BITC on the movement of *S. typhimurium* and its flagella. The results are shown in Figure 5. With the increase in BITC concentration, the motility diameter of *S. typhimurium* on the plate decreased significantly, and the inhibitory effect gradually increased. The results showed that BITC could significantly inhibit the motility of *S. typhimurium* in a concentration-dependent manner.

### 2.6. Effects of BITC on Biofilm Formation of S. typhimurium

**Figure 6:**
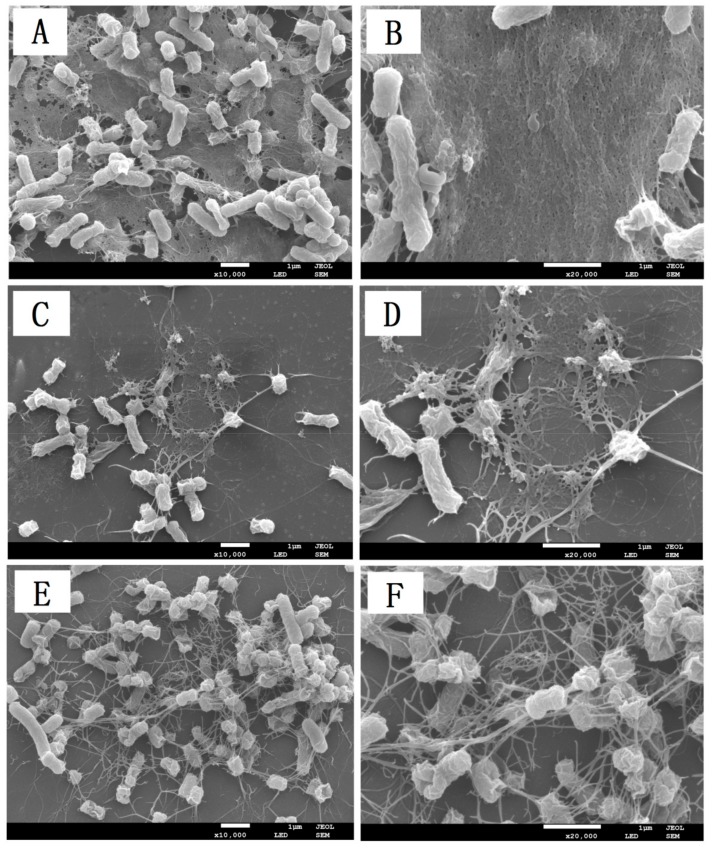
Effects of BITC on biofilm formation of *S. typhimurium* at different concentrations. *S. typhimurium* was added with BITC at 1/4 MIC (**C**,**D**) or 1/8 MIC (**E**,**F**) in 6-well plates and cultured for 36 h. MH instead of BITC was used as negative control (**A**,**B**). The samples were observed under SEM, and the images were collected at 10000 × (**A**,**C**,**E**) and 2000× (**B**,**D**,**F**) magnifications.

There is a close relationship between biofilm formation and bacterial pathogenicity. We can explore and speculate the mechanism of natural bacteriostatic agents by investigating the changes of biofilm formation characteristics in foodborne pathogens. The effect of BITC on biofilm formation of *S. typhimurium* was observed by using scanning electron microscopy. As shown in Figure 6A,B, in the control groups (without BITC treatment), more biofilm was detected adhering to the slides. However, compared to the control group, the samples treated with 1/8 MIC and 1/4 MIC BITC showed significant decreases in the adhesion rate that was, dependent on concentration (Figure 6C–F). In addition, damaged bacteria (shrinkage, corrugation, and rupture) were present on the slides treated with BITC at concentration of 1/4 MIC (Figure 6D), while no damaged bacteria were present on the slides in the control group (Figure 6B). Therefore, it indicated that BITC had a significant inhibitory effect on the biofilm formation of *S. typhimurium*. 

## 3. Discussion

As a common foodborne bacterium, *S. typhimurium* has an important impact on food safety and human health. Therefore, the study of natural bacteriostatic agents for *S. typhimurium* has never stopped. For example, according to Wafa et al., anthocyanin from pomegranate peel extract had good antibacterial activity against *S. typhimurium*, and the MIC was 10.75–12.5 mg/mL [26]. Dihydromyricetin from momordica, as a new bacteriostatic agent, had a significant effect on the oxidative respiration metabolism and membrane integrity of *S. typhimurium* with MIC of 0.625 mg/ml [27]. Compared with the other natural extracts, BITC has a more significant antibacterial effect on *S. typhimurium*, and the MIC is only 250 μmol/L. This highlights that BITC is a good natural product for inhibiting *S. typhimurium*. Moreover, it has been proven by a large number of experimental studies that the antibacterial effect of BITC against foodborne pathogens is of great significance for food safety and clinical research [12,28,29,30].

With the development of genome technologies, the study of *S. typhimurium* by transcriptome analysis is increasing. Das et al. studied transcriptome changes of *S. typhimurium* after treatment with cranberry extract and found that the expression of several pathogenicity-related genes, including *ssaT* on virulence island II, were significantly downregulated [22]. SPI2, which encodes T3SS2, plays an important role in the survival and pathogenicity of *S. typhimurium* in host cells [31,32,33]. As shown in Figure 4, after BITC treatment, the expression levels of virulence-related genes in *S. typhimurium* SPI2, including *ssaT*, *ssaH*, *sseB*, *sseD*, *pagC*, *yscR*, and *sifB*, were significantly decreased. These results are consistent with the previous studies [34,35]. Furthermore, transcriptome technology has also been used to explore the antibacterial mechanism of BITC against *Vibrio parahaemolyticus* and *Staphylococcus aureus*. The results showed that after BITC treatment, the virulence related genes were significantly down regulated, such as *thd*, *fliA*, *fliG,* and *fliI* of *Vibrio parahaemolyticus*; *nuc*, *spa*, *cp8F* of *Staphylococcus aureus* [24,25]. Hence, the application of transcriptome analysis can be significant for exploring bacteriostatic mechanisms.

A large number of studies have demonstrated that the motility of bacteria is related to flagella, which are closely related to the pathogenicity of bacteria; thus, research on the influence of bacteriostatic agents on the motility of bacteria is also extensive [36]. According to Lou et al., eugenol and its nanoemulsion both had a significant effect on the swarming motility and biofilm formation of foodborne bacteria [37]. Pomegranate peel extract has also been shown to have a strong effect on *S. typhimurium* motility [38]. In Figure 5, it is obvious that the swimming and swarming abilities of *S. typhimurium* after BITC treatment were significantly reduced. When the concentration was equal to the MIC, the motility of *S. typhimurium* was completely inhibited. Jitendra et al. [28] have indicated that 125 μg/mL BITC for *S. typhimurium* and 150 μg/mL BITC for *E. coli O157:H7* can significantly inhibit the motility of both bacteria. Selwan et al., and Smriti et al., [39,40] have shown that the motility regulated by flagella is significantly different when the expression of flagella system-related genes is changed. Therefore, we inferred from the transcriptome that the changes in *S. typhimurium* motility after BITC treatment may be due to the downregulation of *yscR* gene expression, but further verification is still needed.

Based on previous studies, biofilms are closely related to the pathogenicity of pathogenic bacteria [8,9]. BITC as main active compound in plant extracts had a significant inhibitory effect on the biofilm formation in bacteria, such as *Escherichia coli* O157:H7 [41] and *Streptococcus mutans* [42]. In Figure 6, it is obvious that, after being treated with BITC, the adhesion of the biofilm was significantly reduced, and *S. typhimurium* was wrinkled and deformed at 1/4 MIC concentration. This conclusion is consistent with the results of Borges et al., and Saleh et al., showing that BITC significantly inhibits *S. typhimurium* growth and changes the integrity of biofilms [15,17]. These results indicate that BITC has significant influence on the biofilm formation characteristics of *S. typhimurium*. In our preliminary studies, we found that the relative electrolysis leakage and extracellular ATP content of *S. typhimurium* increased to varying degrees, while the membrane potential decreased after BITC treatment (data not shown). This suggests that the BITC may cause the cell membrane damage of *S. typhimurium*, and we inferred that the mechanism of BITC on *S. typhimurium* may be related to biofilm as well as cell membrane.

## 4. Materials and Methods

### 4.1. Bacterial Strain and Activation

*Salmonella Typhimurium* ATCC14028 was obtained from China microbial culture preservation center. The frozen *S. typhimurium* stock was inoculated on beef extract peptone medium and cultured at 37 °C for 12 h. Then, 100 μL of bacterial suspension was transferred to 10 mL of the same liquid medium and cultured to a stable growth stage. 1 mL of *S. typhimurium* bacterial suspension was inoculated to 100 mL beef extract liquid medium, simultaneously, BITC was added at subinhibitory concentrations. Bacterial suspension without BITC was used as negative control. After culture for 12 h at 37 °C, bacterial suspension was centrifuged at 8000 rpm for 10 min at 4 °C. The thallus precipitate was used for further studies.

### 4.2. Antimicrobial Tests

The minimum inhibitory concentration (MIC) was determined by broth microdilution. After the dilution of BITC at different multiples, it was added to a sterile 96-hole microporous plate together with the bacterial culture solution for 12 h at 37 °C. The control group was MHB with or without bacterial culture.

### 4.3. RNA Extraction

After treatment with 1/4 MIC BITC, *S. typhimurium* was cultured to a stable growth stage, which was followed by total RNA extraction using RNAprep Pure Cell/Bacterial Kit (Tiangen Biotech, Beijing, China). Total RNA concentration was determined by measuring OD260 nm/OD 280 nm ratio. Agarose gel electrophoresis and a UV transilluminator (Versa Doc, Shanghai, China) were used to examine whether the RNA had been contaminated or degraded. The RNA samples were stored at –80 °C until use.

### 4.4. Building Library Sequencing 

The sequencing library was prepared using the NEBNext®Ultra™ directed RNA library preparation kit for Illumina ® (NEB, Ipswich, MA, USA), which enabled the addition of index codes to the sequence. After the RNA samples passed the test, the ribo-zero kit was used to remove the rRNA and enrich the mRNA. On the basis of single - stranded cDNA synthesized with mRNA as the template, the synthesis of double-stranded cDNA was completed. The cDNA fragments were purified using the AMPure XP system (Beckman Coulter, Beverly, MA, USA), the purified double-stranded cDNA was connected to the sequencing joint and the desired fragment size was selected. Finally, PCR amplification was performed to purify the product and construct the final library. Primer Index (X), Phusion high-fidelity DNA polymerase and universal PCR primers were used for the reaction. The Agilent Biological Analyzer 2100 system was used to evaluate the quality of the product and library. 

### 4.5. Bioinformatic Analysis

Sequence information and quality were evaluated through the establishment of a library and the original sequencing obtained by high-throughput sequencing. To ensure that analysis requirements were met, low quality spliced raw reads were filtered to obtain high quality clean reads for subsequent sequencing analysis. The most commonly used measure of gene expression level was FPKM, the number of fragments per 1000 base length of a gene per million fragments. FPKM could detect the effects of gene length on reading and depth. Volcanic maps were used to estimate the overall distribution of differential genes. During the GO enrichment analysis, we considered *p* < 0.05 as significantly enriched. KEGG is a database that can systematically analyze gene function and genomic information. We used KOBAS software to conduct a holistic study on gene and expression information in the KEGG pathway.

### 4.6. qRT-PCR Validation of Differentially Expressed Genes

To verify transcriptomics results, RNA was purified using the PrimeScript™RT kit and gDNA eraser (TaKaRa, Dalian, China), and cDNA synthesis was performed after genomic DNA was removed. qRT-PCR was performed using SYBR®Premix Ex Taq™II (TliRNaseH Plus) (Takara, Dalian, China). The *16S rRNA* gene was selected as the reference gene, and gene expression calculation was performed using the 2^−ΔΔCt^ method [43]. Primer 5.0 software (PREMIER Biosoft, Palo Alto, CA, USA) was used to design specific primers, as shown in Table 3.

### 4.7. Inhibition of S. typhimurium Motility

According to Sheng et al. [44], swimming and swarming media were prepared. After sterilization and cooling, 500 μL of BITC reserve solution at different concentrations (MIC, 1/2MIC, 1/4MIC, 1/8MIC) was added and mixed evenly. The reversed plate was cooled and dried, and equal amounts of normal saline were added as a negative control. Bacterial solution (3 μL) in the logarithmic growth phase was added to the center, and the diameter of the diffusion circle was measured after being cultured upside down in a constant temperature incubator at 37 °C for 12 h and 48 h to observe the effects of BITC on the swimming and swarming abilities of *S. typhimurium*, respectively.

### 4.8. Scanning Electron Microscopy (SEM)

According to the previously reported method [45], a cover glass was placed in a 6-well microtitration plate, and *S. typhimurium* bacteria and certain nutrients were added for overnight coculture. Different concentrations of BITC were added until the final concentrations reached 1/4 MIC and 1/8 MIC. MH was added to the control group. The slides were subsequently rinsed with PBS several times, soaked in 2.5% glutaraldehyde to fix the biofilms on the slides and then dried with ethanol at concentrations of 50%, 70%, 80%, 90%, and 100%. The finished slides were glued to the table, sprayed with gold and then observed under SEM (JSM6360, JEOL, Tokyo, Japan).

### 4.9. Statistical Analysis

The data were presented as the mean ± standard deviation (n = 3). The difference between the samples were considered statistically significant when *p* < 0.05.

## 5. Conclusions

Research on the antibacterial mechanism of BITC against foodborne pathogens is of great significance for food safety. In this study, transcriptome analysis and phenotypic validation were used for the first time to further explore the expression differences of pathogenicity-related genes. The other important differentially expressed genes as well as proteomics are needed to further verify the mechanism of bacteriostasis of BITC. These results are helpful for studying the antibacterial mechanisms of natural extracts against *S. typhimurium*.

## Figures and Tables

**Table 1:** Summary of RNA-seq alignment.

Sample Name	Raw Reads	Clean Reads	Clean Bases (Gb)	Error (%)	Q20 (%)	Q30 (%)	GC (%)
SC1	24399752	23567734	3.54	0.02	98.46	95.16	53.1
SC2	21013012	20367416	3.06	0.02	98.24	94.74	52.6
SC3	14444786	14083514	2.11	0.02	98.28	94.78	52.89
SQ_BITC1	17951308	17535420	2.63	0.03	98.00	94.15	52.95
SQ_BITC2	15258326	14882480	2.23	0.02	98.12	94.46	54.02
SQ_BITC3	19597206	19162966	2.87	0.02	98.19	94.62	52.94

Q20: percentage of bases with a Phred value > 20; Q30: percentage of bases with a Phred value > 30.

**Table 2:** Data for the differentially expressed genes.

Gene_ID	Gene Name	log^2^FoldChange (SQ_BITC vs. SC)	Pval (SQ_BITC vs. SC)	Padj (SQ_BITC vs. SC)	Significant (SQ_BITC vs. SC)
1248073	*ssaT*	−1.5829	0.0026999	0.48096	DOWN
1249210	*STY2897*	−1.4314	0.0034612	0.48693	DOWN
1248241	*pagC*	−1.234	0.006776	0.68515	DOWN
1248075	*yscR*	−1.4305	0.009399	0.7991	DOWN
1248096	*sseB*	−1.2767	0.014982	1	DOWN
1247852	*sifB*	−1.356	0.01751	1	DOWN
1248087	*ssaH*	−1.3073	0.020987	1	DOWN
1248093	*sseD*	−1.3507	0.022094	1	DOWN

**Table 3:** Primers used to verify gene expression level by quantitative real-time polymerase chain reaction (qRT-PCR).

Gene	Primer	Sequence (5′→3′)
*16S rRNA*	*16S rRNA*-F	GCGGCCCCCTGGACAATGAC
	*16S r* *RNA*-R	TAGCTAAGGAAGCCACGCCT
*s* *saT*	*ssaT*-F	ATCGGTCGGCACAACAAC
	*ssaT*-R	GATGAAGAGCATAAGGGA
*STY2897*	*STY2897*-F	ATGGATGGGTGTCGTGTC
	*STY2897*-R	GAATGGTCGCCTTTACTG
*p* *agC*	*pagC*-F	TACGGCTCTTTTATGGTTGGG
	*pagC*-R	ATCCTGAGTGGAATGTTCTTTA
*y* *scR*	*yscR*-F	ATGTCTTTACCCGATTCGCCTTTG
	*yscR*-R	ACTTGTTGAATACCCAGAGC
*s* *seB*	*sseB*-F	GGTGTTTTGCTTATTCTCCTTA
	*sseB*-R	CATCCATCTCATTTGACTTTTC
*s* *ifB*	*sifB*-F	GCTATGTTGCTTGTTCCCTG
	*sifB*-R	CTTTTCTTTCCTGTTCCTTC
*s* *saH*	*ssaH*-F	AACCATAGCCTGATTTCC
	*ssaH*-R	GCCAACAATAATGCCAGA
*s* *seD*	*sseD*-F	GCTATGTTGCTTGTTCCCTG
	*sseD*-R	GCGGCTTTTCTTTCCTGTTC

## Abbreviations

BITC	benzyl isothiocyanate
DEGs	differential expression genes
FPKMs	Fragments Per Kilobase of exon model per Million mapped reads
GO	Gene Ontology
KEGG	Kyoto Encyclopedia of Genes and Genomes
qRT-PCR	quantitative Real-time Polymerase Chain Reaction
SEM	scanning electron microscopy
